# Inclinometer use in primary total hip arthroplasty does not improve acetabular component positioning: a non-randomized control trial

**DOI:** 10.1186/s42836-024-00258-y

**Published:** 2024-07-05

**Authors:** Kyle Goldstein, Wyatt Tyndall, Michaela E. Nickol, Johannes M. van der Merwe

**Affiliations:** Adult Reconstruction Subdivision, Orthopaedic Division, University of Saskatchewan, Saskatoon, SK S7K0M5 Canada

**Keywords:** Inclinometer, Total hip arthroplasty, Inclination, Abduction angle, Acetabular safe zones

## Abstract

**Introduction:**

Total hip arthroplasty (THA) is a common surgical procedure that aims to relieve pain, improve function, and increase mobility in patients with hip joint pathology. One of the most challenging aspects of THA is to determine the correct angle of the acetabular component’s placement. Intraoperative inclinometers have emerged as a promising tool to obtain accurate measurements of the acetabular component’s inclination. The primary objective of this study was to evaluate the accuracy and efficacy of using intraoperative inclinometers for THA.

**Methods:**

This non-randomized control trial evaluated patients undergoing primary THA. Patients in the inclinometer group had an inclinometer used intraoperatively to measure acetabular component inclination, and patients in the control group had no inclinometer. Inclination and anteversion of the acetabular component were measured on postoperative radiographs.

**Results:**

A total of 223 patients were included in the study. The mean inclination angle of the acetabular cup was significantly higher in the inclinometer group (43.9° vs. 41.5°, *P* < 0.001). This difference was not clinically significant. There was no significant difference in anteversion. There were no significant differences in the number of patients within the safe zones for inclination or anteversion, or in the number of patients experiencing a dislocation. No correlation was found between inclinometer measurement and measured acetabular component inclination. Inclinometer use and body mass index (BMI) were the sole statistically significant factors in determining acetabular component inclination.

**Conclusions:**

This study indicated no current benefit to inclinometer use during primary THA, as measured by inclination, anteversion, and dislocation rate. However, this might be confounded by subtle variations in patient positioning, which may be a strong area of study in the future.

**Supplementary Information:**

The online version contains supplementary material available at 10.1186/s42836-024-00258-y.

## Introduction

Total hip arthroplasty (THA) is a common surgical procedure that aims to relieve pain, improve function, and increase mobility in patients with hip joint pathology. Number of hip arthroplasties performed in the United States is expected to increase by 174% by 2030 [1]. Therefore, improvement in satisfaction will be an important issue surgeons need to address. Multiple factors have been proposed to contribute to the success of a total joint arthroplasty, and the precise placement of components remains a source of significant discussion [2]. Accurate and reliable placement of the acetabular component can be quite challenging but is critical for the success and longevity of the implant [2]. Surgeons use multiple aids to help with accurate placement, including external guides, digital and mechanical protractors, anatomical landmarks, patient positioning, and intraoperative fluoroscopy [2–6]. None of the aids have been shown to be superior in producing accurate and reliable cup placement. In addition, traditional methods can be imprecise and operator-dependent [7–9], while anatomic landmarks can be difficult to identify and accurately recreate [8, 10–12]. External guides are the most commonly employed aid. These guides come with a very low cost, but accurate and reliable placement of an acetabular component, even in experienced hands, can be inconsistent. One proposed reason could be that external guides use a fixed angle of 45° and are influenced by patient habitus and position. Computers and navigation have shown promise to be the most accurate but do come with increased costs, pin site complications, increased expenses, and longer surgical duration [2–6].

The ideal modality is efficient to utilize, cost-effective, easy to master, and, most importantly, is able to produce reliable and precise results. Multiple technological modalities have been developed to help surgeons achieve the aforementioned goals, including navigation, augmented reality, and robotics [3–6]. Unfortunately, each modality does have its own drawbacks, which makes it difficult to show superiority that could lead to global adaptation.

Intraoperative inclinometers have emerged as a promising tool to obtain accurate measurements of the acetabular component’s inclination [8, 13]. Inclinometers are devices which measure angles of slope, elevation, and depression of an object relative to gravitational orientation [2]. Different inclinometers are available. They include the bubble subtype, gravity actuated pendulums attached to the insertion rod, and electronic types. Inclinometers are considered to be more accurate than fixed external guides and are much simpler and cheaper to use than specialized navigation. One study concluded that there was a significant improvement in accuracy in cup placement at the intended inclination angle [2]. Therefore, implementation of inclinometers can be a simple and relatively inexpensive method of improving acetabular cup placement.

The primary objective of this study was to evaluate the accuracy and efficacy of using intraoperative digital inclinometers for acetabular cup placement in THA. Secondary outcomes included dislocation rates among patients as well as assessment of the correlation between inclinometer measurement and acetabular inclination and anteversion.

## Methods

### Participants

This non-randomized control trial was performed between January 2022 and July 2023, inclusive. All patients undergoing primary THA for any indication were included. Patients with previous hip surgery on the operative hip and those with other conditions that might affect acetabular cup placement were excluded.

Patients who received their operation between January and November 2022, inclusive, had the surgery performed without the use of an inclinometer (control group). For those who had surgery between December 2022 and July 2023, an inclinometer was used to measure inclination of the acetabular cup (inclinometer group).

### Operative technique

All patients receiving THA underwent appropriate consent and surgical preparation. They were primary non-complex total hip arthroplasties, to avoid confounding factors. They received appropriate anesthesia, consisting of either a general anesthetic, spinal anesthetic, or both. All operations were performed by the same fellowship-trained adult reconstruction surgeon in addition to a trained surgical assistant and surgical trainees. Patients were positioned in a lateral decubitus position with the affected side upwards and were held with a stable positioner. A Stulburg positioner was used for patients with a body mass index (BMI) less than or equal to 35 kg/m^2^, and a De Mayo positioner for patients with BMI larger than 35 kg/m^2^. A posterior approach was used for all surgeries. When possible, a minimally invasive approach was used. Though various acetabular implants were employed as necessary, the most common implant was the Zimmer Trilogy IT Acetabular System [14]. Acetabular liners were either neutral or elevated, as deemed necessary by the operative surgeon. Various femoral implants were used, both uncemented and cemented, the most common being an uncemented Zimmer ML Taper Femoral Stem [15].

Patients underwent a standardized postoperative course involving attempted discharge from the hospital on postoperative Day 1, and a multi-modal analgesic regimen.

### Inclinometer use

For patients in the inclinometer group, an eOUTIL digital angle gauge (inclinometer) was used intraoperatively to assess inclination during insertion of the acetabular cup. The same inclinometer was utilized for all measurements. The inclinometer was referenced to the operating floor which was considered to be level. During acetabular implant placement, an offset shell inserter was used in tandem with an alignment frame. The magnetic base of the inclinometer was placed on the anterior rod of the alignment frame (Fig. S1, Supplementary Information). The inclinometer measurement was viewed and adjusted accordingly to achieve an inclination angle as near to 40° as reasonably possible.

### Outcome assessment

Primary outcomes were inclination and anteversion angles of the acetabular component. These were measured on non-portable, supine, anteroposterior pelvis radiographs. Measurements were performed using the Intellijoint Surgical Intellijoint View program, as described in the Intellijoint View technique guide (Fig. S2, Supplementary Information) [16]. Two independent observers, who were blinded to patient grouping, performed the measurements. To verify inter-rater reliability, 20 randomly selected patients were measured by both. Following verification of reliability, the remaining patients were randomly divided between the observers, who each measured one-half of the radiographs.

Inclination and anteversion angles were determined to lie within or outside of a pre-defined safe zone in which dislocation is deemed to be least likely. The Lewinnek safe zone was used, established a priori as 30°–50°, inclusive, for inclination and 5°–25°, inclusive, for anteversion [17].

Secondary outcomes included dislocation rate among patients, as assessed through chart and image review, and assessment of correlation between inclinometer measurement and acetabular inclination and anteversion.

### Statistical analysis

Statistical analyses were performed using IBM SPSS Statistics Version 29 [18].

For inter-rater reliability assessment, a two-way mixed intraclass correlation coefficient was calculated. A priori, reliability was defined as poor when the coefficient was less than 0.40, and as fair when it was 0.40–0.59, good 0.60–0.74, and excellent 0.75–1.0 [19].

Scale variables were assessed using an independent-sample *t*-test, and presented as mean and standard deviation (SD). Nominal variables were evaluated using a Chi-Square test and Pearson’s Correlation Coefficient. A correlation analysis was conducted to assess the relationship between inclinometer measurements and acetabular inclination and anteversion. For all tests, statistical significance was defined as a *P* < 0.05.

A linear regression analysis was performed to assess the correlation between various independent variables and measured inclination. A second analysis was undertaken to establish the correlation between the same independent variable and inclination. Statistical significance for each variable was again defined as a *P* < 0.05.

## Results

### Patient characteristics

A total of 223 patients were involved in the study. There were 108 patients in the inclinometer group and 115 in the control group. Patient characteristics are shown in Table 1. There were no significant differences between groups in age, sex, BMI, operative side, or liner type used (neutral or elevated).

**Table 1:** Patient demographics

Demographic	Inclinometer Group (*n* = 108)	Control Group (*n* = 115)	*P*-value
Age, mean ± SD	69.0 ± 10.5	68.7 ± 11.4	0.830
Sex, *n* (%)			
Male	43 (39.8)	57 (49.6)	0.143
Female	65 (60.2)	58 (50.4)	
BMI, mean ± SD	30.7 ± 5.3	31.0 ± 6.3	0.729
Operative Side, *n* (%)			
Left	56 (51.9)	48 (41.7)	0.130
Right	52 (48.1)	67 (58.3)	
Liner, *n* (%)^a^			
Neutral	59 (57.8)	50 (45.5)	0.071
Elevated	43 (42.3)	60 (54.5)	

^a^Six patients in the inclinometer group and 5 patients in the control group did not have liner type recorded

### Inter-rater reliability

Twenty patients were measured by both independent observers to assess inter-rater reliability. Intraclass correlation coefficients for these measurements were 0.882 for inclination and 0.830 for anteversion, both indicating excellent reliability. *P*-values for both scores were < 0.001.

### Primary outcomes

Primary outcomes are listed in Table 2.

**Table 2:** Outcome measurements

Outcome	Inclinometer Group (*n* = 108)	Control Group (*n* = 115)	*P*-value
Inclination, mean ± SD (°)	43.9 ± 4.7	41.5 ± 6.7	< 0.001
Safe Inclination^a^, n (%)	96 (88.9)	98 (85.2)	0.415
Anteversion, mean ± SD (°)	24.8 ± 6.7	24.0 ± 6.8	0.447
Safe Anteversion^b^, *n* (%)	62 (57.4)	63 (54.8)	0.693
Safe Inclination and Safe Anteversion, *n* (%)	58 (53.7)	54 (47.0)	0.314
Dislocation, *n* (%)	0 (0)	2 (1.7%)	0.169

^a^Safe inclination zone defined as 40 ± 10 (°)

^b^Safe anteversion zone defined as 15 ± 10 (°)

Inclination and anteversion angles were measured on postoperative anteroposterior supine radiographs. The inclinometer group had a mean inclination of 43.9°, while the control group registered a mean inclination of 41.5°. This difference was statistically significant (*P* < 0.001). The inclinometer group had a mean anteversion of 24.8°, and the control group yielded a mean anteversion of 24.0°. This difference was not statistically significant (*P* = 0.447).

The measured inclination and anteversion were evaluated to establish if they were within the pre-defined safe zone. Ninety six patients (88.8%) in the inclinometer group were within the inclination safe zone, compared to 98 (85.2%) in the control group. This difference was not statistically significant (*P* = 0.415). Sixty two (57.4%) patients in the inclinometer group were in the anteversion safe zone, compared to 63 (54.8%) patients in the control group. This different was not statistically significant either (*P* = 0.693). Fifty eight patients (53.7%) in the inclinometer group were in the safe zone for both inclination and anteversion, while 54 patients (46.9%) in the control group were within both safe zones. This difference was not statistically significant (*P* = 0.314).

### Other outcomes

No patients in the inclinometer group experienced a dislocation, while there were 2 hip dislocations in the control group. This difference was not statistically significant (*P* = 0.169). One dislocation was posterior and occurred in the recovery room, while the second was anterior in the early postoperative period due to non-adherence to hip precautions.

**Fig. 1:**
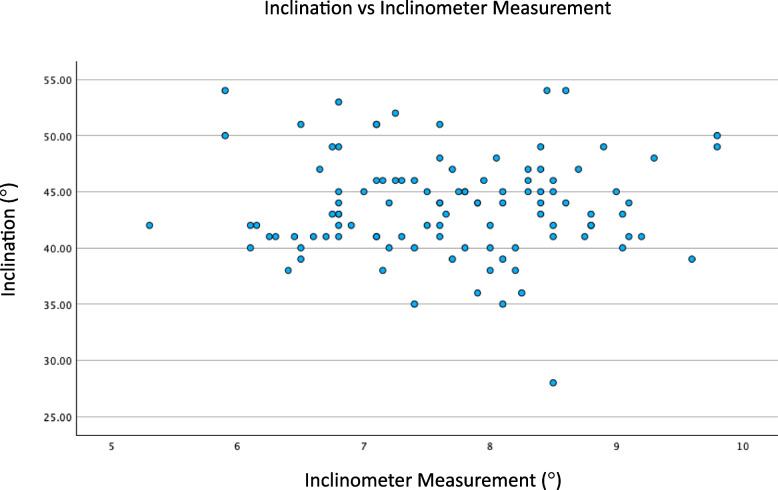
Scatter plot of inclination vs. inclinometer measurement

**Fig. 2:**
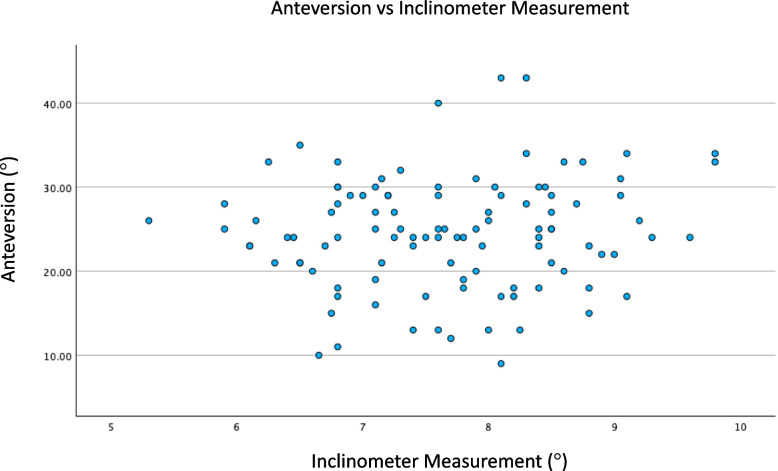
Scatter plot of anteversion vs. inclinometer measurement

Analysis was performed to determine if inclinometer measurement correlated with measured inclination and anteversion. Scatter plots are displayed in Figs. 1 and 2. There was no correlation between inclinometer measurement and inclination (*P* = 0.374). Similarly, there was no correlation between inclinometer measurement and anteversion (*P* = 0.306).

A linear regression analysis was performed to assess for correlation between various independent variables and inclination. The analysis revealed statistically significant differences (*P* = 0.003). Statistically significant independent variables included inclinometer use and BMI (*P* = 0.002 and *P* = 0.022, respectively). Both inclinometer use and higher BMI were correlated with higher inclination. There was no statistically significant correlation with sex, age, operative side, or liner type.

A second linear regression analysis revealed no statistically significant correlation between the same independent variables and anteversion.

## Discussion

Acetabular cup placement is very challenging, and even experienced adult reconstruction surgeons struggle to place the cup in an accurate and reproducible manner [2, 11, 12]. Placement of the acetabular component is associated with long-term outcomes and patient satisfaction [2]. Technologies have been introduced into orthopaedic surgery to help surgeons achieve precise and consistent acetabular cup placement during THA. Technology, however, comes with an increased cost and learning curve which precludes surgeons from using it readily [3–6]. Inclinometers, conversely, are easy to use without a learning curve, do not have significant costs, and can be used repeatedly. Multiple studies have shown improvement in acetabular cup placement with the use of an inclinometer [13, 20–24]. Contrarily, a randomized controlled trial of 100 THA participants did not show a clear benefit of using an inclinometer [23].

This study demonstrated a statistically significantly higher inclination angle for patients in the inclinometer group (43.9°) compared to the control group (41.5°). This differed from another study that showed a reduction in inclination angles with the use of the inclinometer [13]. The mean inclination angles in that article were 42.9° for the inclinometer group and 46.5° for the control group. This was similar to a previously published study, which demonstrated an inclination angle of 42.2° in the inclinometer group compared to 44.4° in the control group [22]. It is unclear why the inclination angles in the present study were increased with the inclinometer usage. One potential reason may be underestimation of the true depression of inclination during inclinometer use by the operating surgeon. The surgeon used 7°–9° on the inclinometer to judge inclination angle for a goal of 42°. Literature review indicated surgeons should rather target 30°–35° when using the external guide, relative to the floor. This will accommodate for the adducted and internally rotated pelvis in patients operated on in the lateral decubitus position [12, 23]. By only using 7°–9° of depression on the inclinometer, the inclination angle would be greater compared to using the external guide. It is important for surgeons to document their radiological inclination amounts and correlate them with the intraoperative inclinometer values to determine which value will reproduce the correct inclination.

There were no statistically significant findings between the groups in terms of inclination safe zones. This was similar to another study which found a low percentage of patients outside the inclination safe zone for both groups [23]. Interestingly, this was different from two other studies that demonstrated that patients where an inclinometer was used were statistically more likely to be placed inside the Lewinnek safe zones [13, 25]. A further randomized controlled trial of 270 participants demonstrated that the digital inclinometer was most accurate for cup placement, with an inclination angle of 35° ± 2.5° in 88% of the cases [21]. In a cadaver study, use of inclinometer resulted in accurate placement of the acetabular component in the inclination safe zones in 100% of the cases compared to visuospatial perception [22]. The reason why we did not find a difference in cup placement in the safe zones between the two groups could be explained by the fact that placement of the cup was not solely determined by the use of inclinometer. Intraoperative landmarks, including the transverse acetabular ligament and relation of the cup and the native acetabulum at the 12 o’clock position as templated preoperatively, contributed to cup placement. Secondly, all surgeries were performed by a high-volume adult reconstruction surgeon with more than 10-year experience.

The number of patients with inclination angle within the anteversion safe zone for both groups was quite low, with 57.4% in the inclinometer group and 54.8% in the control group. This can be explained by the senior author only performing the posterolateral approach, with an anteversion goal of 25° ± 5°, rather than the typical 5°–25° [17].

Two patients (0.8%) experienced a dislocation. Both of the patients developing dislocation were in the control group. With the first patient, a hip dislocation occurred in the recovery room. He was immediately revised to an elevated rim liner and sustained no subsequent dislocations. The second patient did not follow the hip precautions immediately after surgery and slept prone and, as a result, developed a subsequent anterior hip dislocation. He was also successfully treated with a closed reduction with no further instability episodes. In addition, of the two patients who suffered from a hip dislocation, one was within the safe zone for both inclination and anteversion and the other was within the safe zone for inclination but outside the anteversion safe zone. This implies that acetabular cup placement is not solely responsible for dislocations but that instability is a multifactorial issue. However, the results should be interpreted with caution given that there was a very low number of dislocations with a relatively short follow-up.

These primary outcomes appeared to indicate no benefit to using an inclinometer during primary THA, though the lack of correlation between inclinometer measurements and inclination and anteversion warrants discussion. As the inclinometer itself is a reliable tool, lack of correlation indicates other confounding variables. The most obvious of these variables is patient positioning. Although all patients were positioned in the lateral decubitus position and held with identical retractors, their angle of positioning was not measured. Ideally, patients should be placed in an exact lateral position, though this is likely unachievable. This is further supported by the correlation between higher BMI and higher inclination. Other studies have also demonstrated a correlation between hip circumference and acetabular cup inclination [25]. Patients with larger body habitus and additional tissue are more difficult to be placed in a perfect lateral decubitus position, thus increasing variation in acetabular cup placement. Acetabular cup inserters may also be influenced by the extra adipose tissue, which can lead to difficulty in placement of the acetabular component as well as higher forces needed on the retractors with subsequent change in pelvis rotation. This study implied that subtle positional variation plays a large role in acetabular inclination and anteversion that cannot be compensated with more accurate measurement during component placement.

The study does have limitations. The current study had low external validity. All patients were operated on in a tertiary center by a high-volume adult reconstruction surgeon. The results might not be generalizable to the broader population. It, however, might be valuable for high-volume and low-volume surgeons to help with placement of the acetabular component. Having a concrete number displayed during acetabular cup placement may instill confidence in placing the cup compared to visuospatial placement. Moreover, this was a non-randomized trial, which could mean that results obtained from this study might be due to the differences between the two groups rather than the intervention. This could lead to selection bias where certain characteristics could influence who is included in each group. This can make it harder to draw conclusions about cause and effect. However, there were no statistically significant differences in patient demographics between groups. To overcome these limitations, future randomized controlled trials are needed to assess patient positioning and acetabular cup placement during primary THA.

## Conclusions

This study indicated no current benefit to inclinometer use during primary THA, as measured by inclination, anteversion, and dislocation rate. However, this is likely confounded by subtle variations in patient positioning, which may be a strong area of study in the future.

### Supplementary Information

Supplementary Material 1: Fig. S1. Demonstration of inclinometer use. Fig. S2. Demonstration of measurement of acetabular inclination and anteversion.

## Data Availability

All the raw data and materials described in the manuscript are available upon requests to any scientist wishing to use them for non-commercial purposes.
