# Supplementary material for: Inclinometer use in primary total hip arthroplasty does not improve acetabular component positioning: a non-randomized control trial
Source: Arthroplasty. 2024 Jul 5;6:41. doi: 10.1186/s42836-024-00258-y (PMC11229020; doi:10.1186/s42836-024-00258-y)
Supplement: Supplementary file 1 — Supplementary Material 1: Fig. S1. Demonstration of inclinometer use. Fig. S2. Demonstration of measurement of acetabular inclination and anteversion. [file 42836_2024_258_MOESM1_ESM.docx]

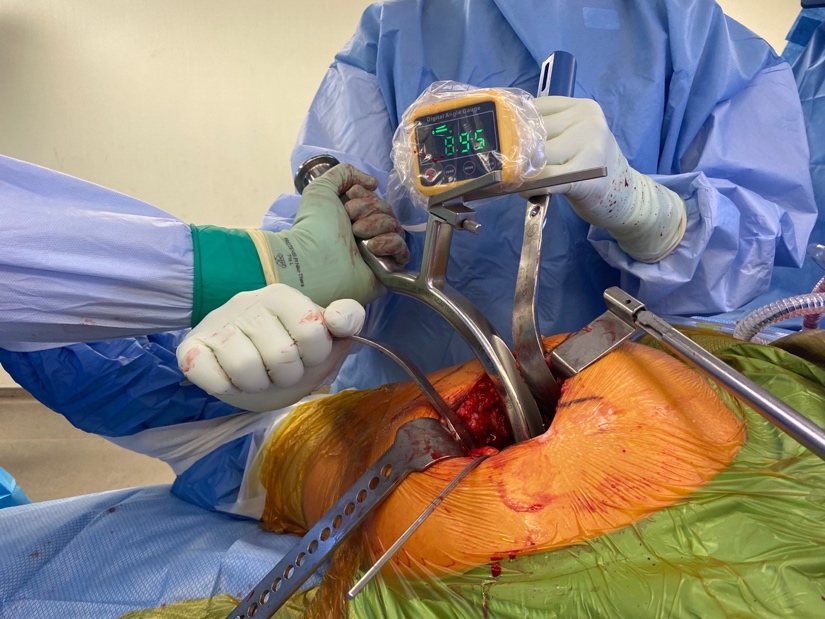


**Fig. S1** Demonstration of inclinometer use.


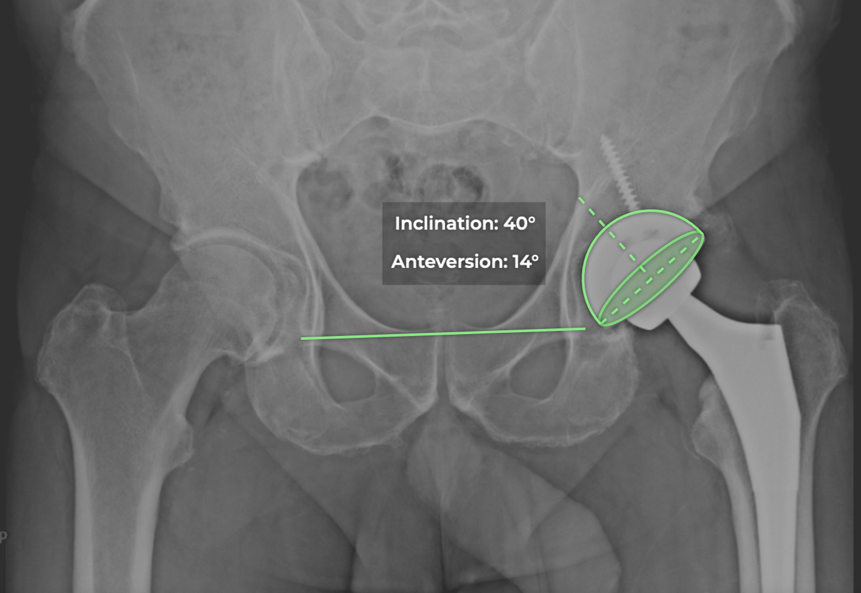


**Fig. S2** Demonstration of measurement of acetabular inclination and anteversion.
